# *Aronia melanocarpa* polysaccharide ameliorates inflammation and aging in mice by modulating the AMPK/SIRT1/NF-κB signaling pathway and gut microbiota

**DOI:** 10.1038/s41598-021-00071-6

**Published:** 2021-10-18

**Authors:** Yingchun Zhao, Xinglong Liu, Yinan Zheng, Wencong Liu, Chuanbo Ding

**Affiliations:** 1 College of Chinese Medicinal Materials, Jilin Agricultural University, Changchun, 130118 China; 2 National & Local Joint Engineering Research Center for Ginseng Breeding and Development, Changchun, 130118 China

**Keywords:** Drug discovery, Immunology, Health care, Risk factors, Signs and symptoms

## Abstract

*Aronia melanocarpa* is a natural medicinal plant that has a variety of biological activities, its fruit is often used for food and medicine. *Aronia melanocarpa* polysaccharide (AMP) is the main component of the *Aronia melanocarpa* fruit. This research evaluated the delay and protection of AMP obtained from *Aronia melanocarpa* fruit on aging mice by d-Galactose (D-Gal) induction and explored the effect of supplementing AMP on the metabolism of the intestinal flora of aging mice. The aging model was established by intraperitoneal injection of D-Gal (200 mg/kg to 1000 mg/kg) once per 3 days for 12 weeks. AMP (100 and 200 mg/kg) was given daily by oral gavage after 6 weeks of D-Gal-induced. The results showed that AMP treatment significantly improved the spatial learning and memory impairment of aging mice determined by the eight-arm maze test. H&E staining showed that AMP significantly reversed brain tissue pathological damage and structural disorders. AMP alleviated inflammation and oxidative stress injury in aging brain tissue by regulating the AMPK/SIRT1/NF-κB and Nrf2/HO-1 signaling pathways. Particularly, AMP reduced brain cell apoptosis and neurological deficits by activating the PI3K/AKT/mTOR signaling pathway and its downstream apoptotic protein family. Importantly, 16S rDNA analysis indicated the AMP treatment significantly retarded the aging process by improving the composition of intestinal flora and abundance of beneficial bacteria. In summary, this study found that AMP delayed brain aging in mice by inhibiting inflammation and regulating intestinal microbes, which providing the possibility for the amelioration and treatment of aging and related metabolic diseases.

## Introduction

Aging is a degenerative disease affecting the functions of living organisms and their related nerves^1^. As an inevitable physiological process, aging causes gradual loss of body functions and results in many age-related diseases, including diabetes, cognitive impairment, cancer, liver injury, Parkinson's disease and atherosclerosis^2,3^. Both developed and developing countries have aging populations due to scientific and technological advancement. Therefore, delaying aging has become a focus of many studies^4^. Anti-aging research mainly focuses on inhibiting telomere shortening, resisting lipid peroxidation, scavenging free radicals, regulating immune endocrine secretions, and reducing DNA damage and autophagy^5–9^. Adenosine 5′-monophosphateactivated protein kinase (AMPK) is a key molecule in the regulation of biological energy metabolism and is important in regulating cell growth, proliferation, survival and energy metabolism^10–12^. AMPK is involved in regulating a series of age-related signaling pathways such as SIRT1, NF-κB and p53, which are involved in the regulation of mammalian cell senescence^13^. Silencing message regulator 2-related enzyme 1 (SIRTUIN 1) is a class of NAD^+^-dependent histone deacetylases found in living bodies. SIRT1 is involved in cell survival, apoptosis, stress resistance, inflammation and other physiological activations. The activation of SIRT1 is an important reason for the extension of biological life-span, and it affects the anti-stress ability of cells by directly regulating p53 and NF-κB signaling pathways^14,15^. The regulatory function of AMPK in energy metabolism and the direct or indirect regulation of the above signaling pathways are important, thus it is necessary to study the relationship between AMPK and age-related signaling pathways to find the possible mechanisms of aging.

The d-Galactose (D-Gal)-induced aging model has gradually been used to study the aging mechanism since the long-term administration of D-Gal is known to accelerate the aging of rodents^16^. Excessive D-Gal in the body can cause an accumulation of reactive oxygen species (ROS) in the brain and inhibit the activities of antioxidant enzymes such as catalase (CAT), superoxide dismutase (SOD), and glutathione (GSH)^17^. It can destroy the redox balance defense system and lead to oxidative stress injury^18,19^. Importantly, D-Gal-induced brain aging can cause mitochondrial dysfunction and lead to the decline in cognitive ability by inducing inflammatory injury, cell apoptosis, and the reduction of brain-derived neurotrophic factors^20^. Imbalance of the antioxidant defense system plays an important role in the process of aging^21^; hence, maintaining the maintaining the dynamic balance of redox may be a useful treatment approach to delay D-Gal-induced aging^22^.

*Aronia melanocarpa* is native to North America, and it has been introduced and cultivated in Northeast China on a large scale presently^23^. In September 2018, *A. melanocarpa* was approved as a novel food by the National Health Council of China. It is mainly used for production of fruit juices, jams, sauces, fruit teas, dietary supplements and wines. It is also used as a natural source of food coloring and as an anti-hypertensive, anti-atherosclerotic drug in Russia and Eastern European countries^24–27^. *A. melanocarpa* is rich in flavonoids, polyphenols, polysaccharides, organic acids, dietary fiber and other nutrients. Polysaccharides are active in anti-tumor, hepatoprotective, anti-inflammatory, anti-viral, anti-oxidant and anti-microbial^28,29^. Present studies also find that polysaccharides in traditional chinese medicine are used in anti-aging, such as polysaccharides in *Angelica*, *Lycium barbarum*, and *Astragalus*. Mechanism studies show that they work by scavenging free radicals^30–32^, affecting the length of telomeres at the end of chromosomes, and regulating the body's immune system^33–35^. In this study, animal behavior experiments were used to observe the effects of AMP on spatial learning and the memory abilities of D-Gal-induced aging mice. We also explored the mechanism of AMP on NLRP3 inflammasome by the AMPK/SIRT1/NF-κB signaling pathway. The 16S rDNA showed that the intestinal flora of the feces of each group of mice were significantly different, which suggests that the difference in our intestinal flora differences are important in aging.

## Results

### Monosaccharide composition of AMP

**Figure 1:**
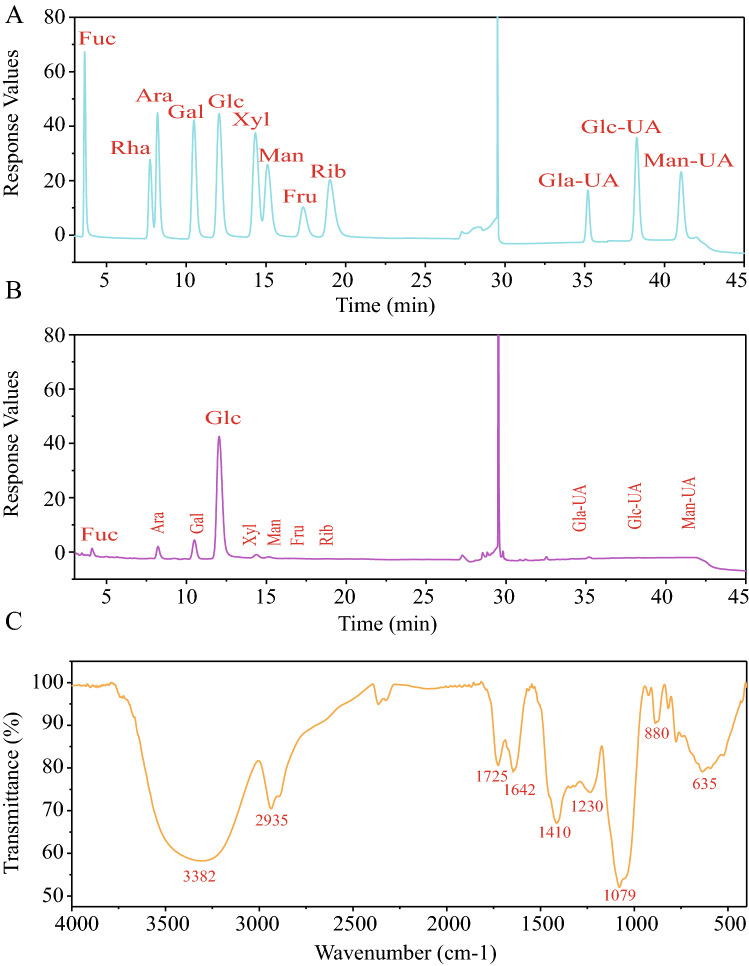
Analysis of the structure of AMP. (**A**) The standard monosaccharides. (**B**) The monosaccharide composition. **(C)** FI-IR analysis of *A.* melanocarpa polysaccharide. The IR spectra was analyzed on JASCO FT/IR-620 spectrometer (http://www.jasco.co.jp/).

Purified polysaccharide was obtained by DEAE-52, and the monosaccharide composition showed that AMP was composed of Fuc (0.14%), Rha(0.73%), Ara (7.14%), Gal (10.61%), Glc (76.16%), Xyl (2.31%), Man (1.25%), Gal-UA (1.43%), Glc-UA (0.16%), and Man-UA (0.07%) (Fig. 1A,B). Glc was the main component, and the uronic acid total ratio was 1.66, while the peak before uronic acid was the solvent peak.

### FT-IR spectra analysis of AMP

The characteristic structure of AMP was shown in Fig. 1C. The 3600–3200 cm^−1^ peak was the stretching vibration absorption peak of –OH, and the absorption peak in this region was the characteristic peak of sugars. The details were as follows: 3382 cm^−1^ was the O–H stretching vibration absorption peak, which is the characteristic peak of sugars. The absorption peak at 2933 cm^−1^ was attributed to the C–H stretching vibration^30^. The absorption peak at 1650 cm^−1^ was attributed to C=O stretching vibration^32^. The absorption peak at 1556 cm^−1^ was attributed to C=O asymmetric stretching vibration^34^. There was an absorption peak at 1402 cm^−1^, which was attributed to the C–O stretching vibration. There was an absorption peak at 1029 cm^−1^, which was attributed to O–H variable angle vibration. Several weak peaks at 1000–800 indicated that α and β glucosides were connected to form pyranose rings^35^.

### AMP improves the general condition of mice

The initial and final body weights of the mice were recorded and the weight gain rate was analyzed. As shown in Table 1. Compared with the normal group, the body weight in model group had an extremely significant effect (*p* < 0.01). The AMP-H group was significantly different compared with the model group (*p* < 0.05). Data showed that more than D-Gal, AMP treatment may affect the weight of mice. Moreover, the organ index of mice was recorded in Table 2, including mouse heart, liver, spleen, kidney, and brain. The results showed that AMP effectively improved the organ index of mice induced by D-Gal, and the improvement effect of each organ was different**.**

**Table 1:** Effect of AMP on the body weight of aging model mice.

Group	Dosage (mg/kg)	Initial weight (g)	Final weight (g)	Growth rate (%)
Normal	–	33.21 ± 1.54	44.11 ± 2.09	33.09 ± 9.17
D-Gal	200–1000	33.18 ± 0.59	40.13 ± 2.1**	20.99 ± 6.67**
AMP-L	100	33.28 ± 0.70	41.76 ± 2.5	25.53 ± 7.76
AMP-H	200	33.18 ± 0.59	42.75 ± 1.46^#^	28.91 ± 5.68^#^

Values represent the mean ± S.D., n = 10. *p < 0.05 or **p < 0.01 vs. normal group; ^*#*^*p* < 0.05 or ^*##*^*p* < 0.01 vs. D-Gal group. The table was drawn using Microsoft Excel 2016 version 3.3.2.13 (https://www.microsoft.com/es-cl/microsoft-365).

**Table 2:** Effect of AMP on the organ index of aging mice.

Group	Dosage (mg/kg)	Organ index (mg/g)				
		Heart	Liver	Spleen	Kidney	Brain
Normal	–	0.59 ± 0.03	4.67 ± 0.38	1.7 ± 2.19	1.75 ± 0.09	1.26 ± 0.07
D-Gal	200–1000	0.43 ± 0.03*	3.94 ± 0.25**	0.24 ± 0.03**	1.37 ± 0.09**	0.98 ± 0.23**
AMP-L	100	0.49 ± 0.09^#^	3.96 ± 0.24	0.32 ± 0.12	1.49 ± 0.18	1.21 ± 0.16^##^
AMP-H	200	0.52 ± 0.08^##^	4.31 ± 0.32^##^	0.53 ± 0.63	1.59 ± 0.18^##^	1.25 ± 0.13^##^

Values represent the mean ± S.D., n = 10. *p < 0.05 or **p < 0.01 vs. normal group; ^*#*^*p* < 0.05 or ^*##*^*p* < 0.01 vs. D-Gal group. The table was drawn using Microsoft Excel 2016 version 3.3.2.13 (https://www.microsoft.com/es-cl/microsoft-365).

**Figure 2:**
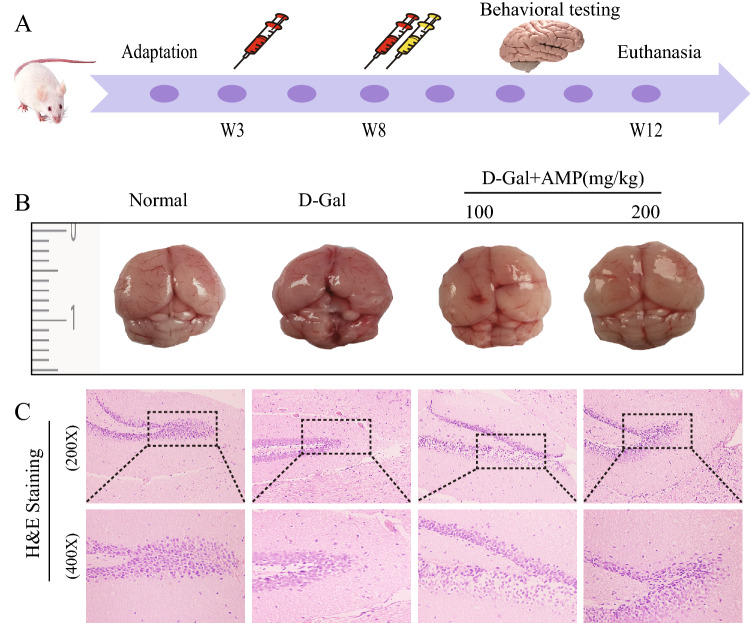
AMP improves the general condition of mice. (**A**) Experimental design of the D-Gal-induced liver fibrosis model in mice. (**B**,**C**) AMP improved the appearance of the whole brain of aging mice and H&E staining showed the pathological damage of the hippocampus. Images were obtained using Canon EOS 1600D\u002F 200D II (http://www.canon.com.cn/), and processed with Adobe Photoshop CC 2018 (https://www.adobe.com/products/photoshop.html). The drawing was created using Adobe Illustrator CS 11.0 (https://www.adobe.com/cn/products/illustrator.html). The stained sections were collected by light- microscope Leica DM750 (https://www.leica-microsystems.com.cn/cn/products/light-microscopes/).

Mice in the normal group had smooth fur and were mentally active, and mice in the D-Gal group were curled up, their fur was sparse and there was no light, and their mental state was poor. The AMP treatment group had better fur and spirit than the D-Gal group. From the appearance of the brain of the mice, the D-Gal group had bled spots on the surface and the brain tissue structure of mice was blurred (Fig. 2B). The AMP treatment group seemed better than the D-Gal group. To explore the pathological conditions of the brain, we analyzed the pathological damage to the hippocampal dentate gyrus under 200 and 400 magnifications (Fig. 2C). Compared with the normal group, the granular cells in the D-Gal group were arranged irregularly, the shape of the nucleus changed, the nucleus was separated from the cytoplasm, and the cell shape was incomplete, indicating cell senescence^33,36^. The AMP treatment groups significantly improved cell morphology and arrangement, especially in the AMP-H dose group.

### AMP improves D-Gal-induced spatial learning and memory

**Figure 3:**
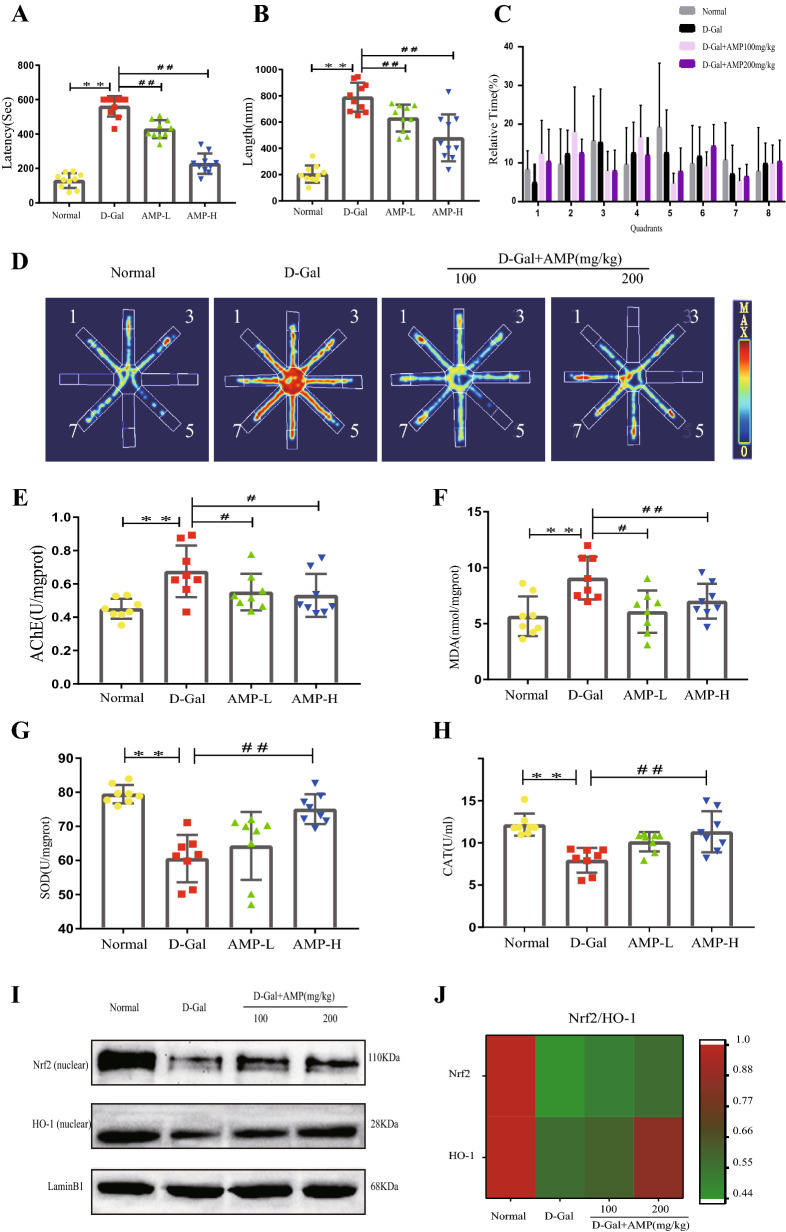
AMP improves D-Gal-induced spatial learning and memory. (**A**,**B**) Latent period and track length of mice in the maze. (**C**) Percentage of time spent by mice in each quadrant. (**D**) Action trajectory diagram of mice in the maze. (**E**) AchE level in mouse brain tissue. (**F**–**H**) The brain levels of MDA, SOD, and CAT in D-Gal-induced senescence. (**I**,**J**) Nrf2 and HO-1 protein expression and heat map analysis of Nrf2 and HO-1 protein expression levels. Data are expressed as the mean ± standard deviation (S.D), n = 10. *p < 0.05 or **p < 0.01 vs. normal group; ^*#*^*p* < 0.05 or ^*##*^*p* < 0.01 vs. D-Gal group. The figures were created by using GraphPad Prism Software version 6.04 (https://www.graphpad.com/). Chemical imaging was collected using Bio-Rad Imaging System version VersaDoc 3000 (https://www.bio-rad.com/). The heatmap was constructed using the online tool Morpheus (https://software.broadinstitute.org/morpheus/). Trajectory imaging was taken using Thermal Imaging Analysis software version RMT-100 (https://tmvmc.com/).

The hippocampus plays a key role in navigation and spatial memory^33,37–39^. To study the spatial memory function of mice in each group, we conduct an eight-arm maze test, which accurately and sensitively reflect the spatial learning and memory function of the brain. This test is an important experimental method used to detect the spatial memory of rodents^40–42^. The results showed that the movement distance and latency of mice in the D-Gal groups were longer, and AMP treatment significantly improved this phenomenon (Fig. 3A–D). However, there was no significant relationship between the latency of the mice in each quadrant. To verify this result, we performed an AchE test of mouse brain tissue (Fig. 3E). The D-Gal group was significantly higher than the normal group, and it was reduced after AMP treatment. The above results all showed that AMP significantly improved the aging phenomenon caused by D-Gal.

### AMP improves D-Gal-induced oxidative stress

Because oxidative stress plays a key role in the aging process, we measured related indicators in brain tissue, such as MDA, SOD, and CAT. The results showed the levels of SOD and CAT in the D-Gal group were significantly reduced, while the MDA was the opposite (Fig. 3F–H). The AMP treatment group significantly improved the situation, reduced the MDA level (*p* < 0.01 or *p* < 0.05), and increased the SOD and CAT levels (*p* < 0.01). These results demonstrated that AMP had a protective effect against oxidative stress induced by D-Gal.

To further explore the antioxidant effect of AMP on aging mice, the Nrf2/HO-1 signaling pathway was analyzed by western-blotting. The results showed that AMP significantly up-regulated the expression of Nrf2 and HO-1 nucleoprotein in the D-Gal group (*p* < 0.01) (Fig. 3I,J). The above results all indicated that AMP improved D-Gal-induced oxidative stress damage.

### AMP inhibits the production of NLRP3 inflammasome through AMPK

**Figure 4:**
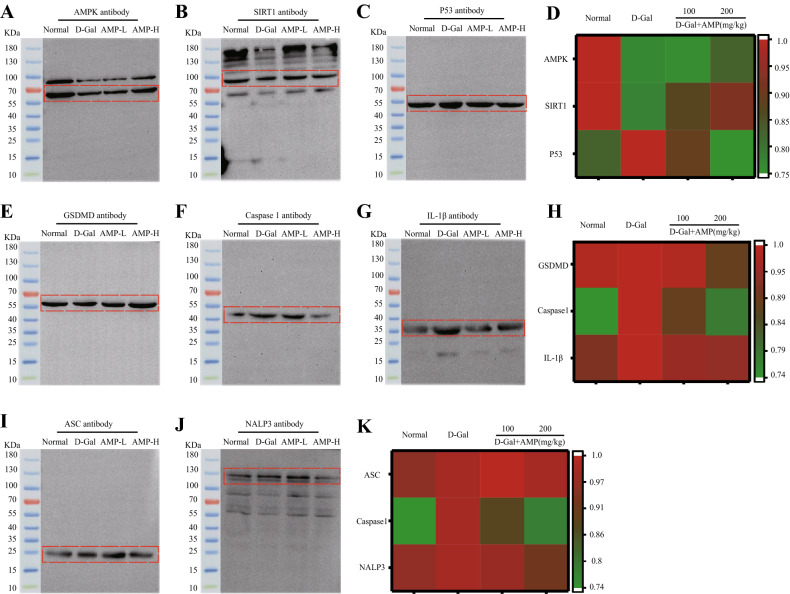
AMP improves D-Gal-induced oxidative stress. (**A**–**D**) AMPK, SIRT1, and P53 proteins expression, and heat map analysis of AMPK signaling pathway expression levels. (**E**–**K**) Inflammasome proteins expression of GSDMD, Caspase 1, IL-1β, ASC, and NALP3, and heat map analysis of inflammasome protein expression levels. Data are expressed as the mean ± standard deviation (S.D), n = 10. *p < 0.05 or **p < 0.01 vs. normal group; ^*#*^*p* < 0.05 or ^*##*^*p* < 0.01 vs. D-Gal group. Chemical imaging was collected using Bio-Rad Imaging System version VersaDoc 3000 (https://www.bio-rad.com/). The heatmap was constructed using the online tool Morpheus (https://software.broadinstitute.org/morpheus/).

The circulatory regulation mechanism between AMPK and SIRT1 reinforces the important role of energy metabolism balance in the aging process. SIRT1 participates in the regulation of the aging process by reducing the activity of the P53 gene^14,15^. Therefore, we tested the aging-related proteins, and the results were shown in Fig. 4A–D. The expressions of AMPK and SRIT1 in the model group were significantly less, while P53 was the opposite.

To determine if AMP has an anti-pyroptotic effect on D-Gal-induced senescence, we detected the levels of related proteins in the classical pyroptotic pathway mediated by Caspase-1. The protein expressions of GSDMD, Caspase-1, and IL-1β in the D-Gal group were all increased, and the AMP treatment group could change this phenomenon (Fig. 4E–H). The occurrence of pyrolysis is often accompanied by the accumulation of inflammasomes. To further explore the molecular mechanism of AMP against pyrolysis, we focused on the expression of NLRP3 and ASC proteins. Compared with the normal group, the protein content of NLRP3 and ASC in the D-Gal group increased, and their expression decreased after AMP treatment (Fig. 4I–K). All of the results indicated that AMP alleviated D-Gal-induced pyrolysis and thereby delayed the aging state of mice.

### AMP reduces D-Gal-induced inflammation

**Figure 5:**
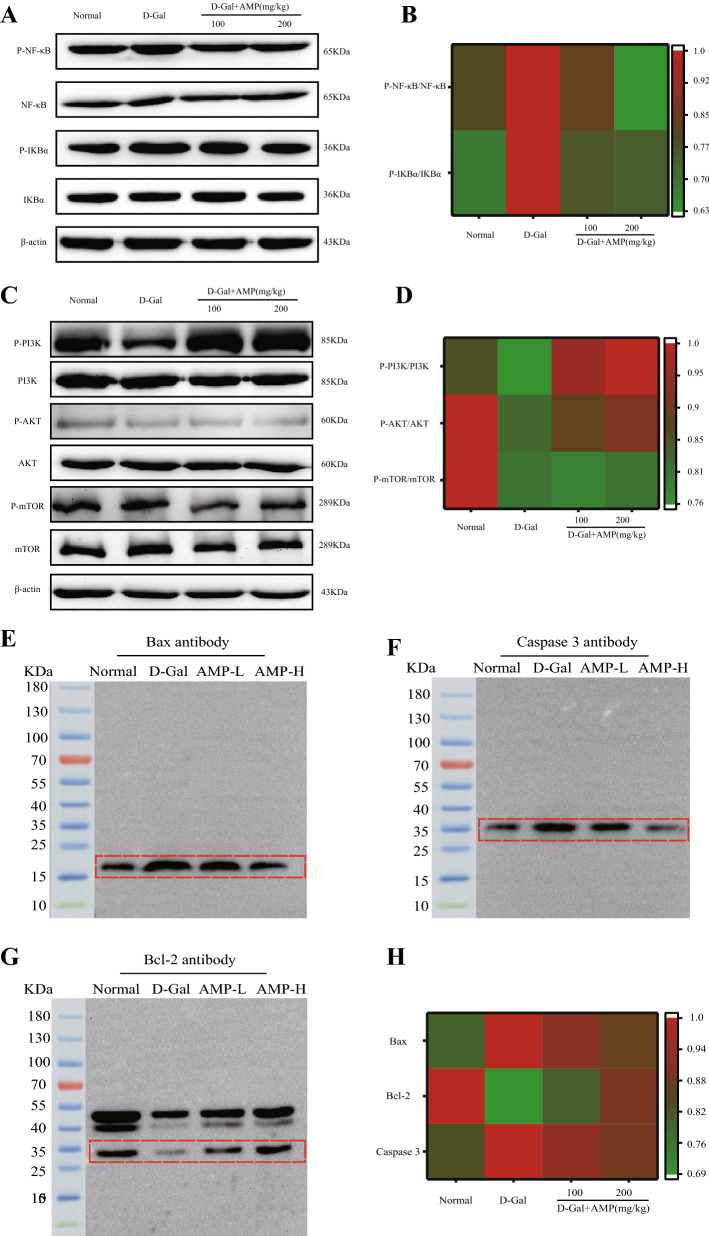
AMP exerts an anti-aging effect in mice by regulating NF-κB-mediated inflammation and PI3K/AKT/mTOR anti-apoptotic pathway. (**A**,**B**) The proteins expression of NF-κB and IκB-α in mice of brain tissue, and heat map analysis of NF-κB signaling pathway expression protein levels. (**C**–**H**) The proteins expression of p-PI3K/PI3K, p-AKT/AKT, p-mTOR/mTOR, Bax, Caspase 3 and Bcl-2 in mice of brain tissue, and heat map analysis of PI3K/AKT/mTOR and its downstream protein levels. Data are expressed as the mean ± standard deviation (S.D), n = 10. *p < 0.05 or **p < 0.01 vs. normal group; ^*#*^*p* < 0.05 or ^*##*^*p* < 0.01 vs. D-Gal group. Chemical imaging was collected using Bio-Rad Imaging System version VersaDoc 3000 (https://www.bio-rad.com/). The heatmap was constructed using the online tool Morpheus (https://software.broadinstitute.org/morpheus/).

To explore whether AMP had an anti-inflammatory effect on D-Gal-induced senescence, the levels of related proteins were determined in the NF-κB classic inflammatory pathway. The use of D-Gal increased the expression of the phosphorylated protein of NF-κB and its upstream regulator IκB (*p* < 0.01). The AMP treatment group could significantly inhibited the high expression of NF-κB and blocked the increase in the expression of the upstream regulatory factor IκB-α phosphorylated protein (*p* < 0.01) (Fig. 5A,B). These results indicated that AMP improved D-Gal-induced aging symptoms through anti-inflammatory effects.

### AMP reduces D-Gal-induced apoptosis through PI3K/AKT pathway

We performed PI3K/AKT-related proteins including downstream apoptotic proteins Bax, Bcl-2, and Caspase3. The AMP treatment group significantly increased the expression of P-PI3K, P-AKT, and P-mTOR proteins compared with the D-Gal group (*p* < 0.05 or *p* < 0.01) (Fig. 5C–H). Furthermore, for its downstream apoptotic proteins, AMP significantly reduced the expression of Bax and Caspase3 proteins, while the results of Bcl-2 were the opposite (*p* < 0.05 or *p* < 0.01). These results indicated that AMP inhibited D-Gal-induced apoptosis.

### AMP improves D-Gal-induced aging by regulating the intestinal flora of mice

**Figure 6:**
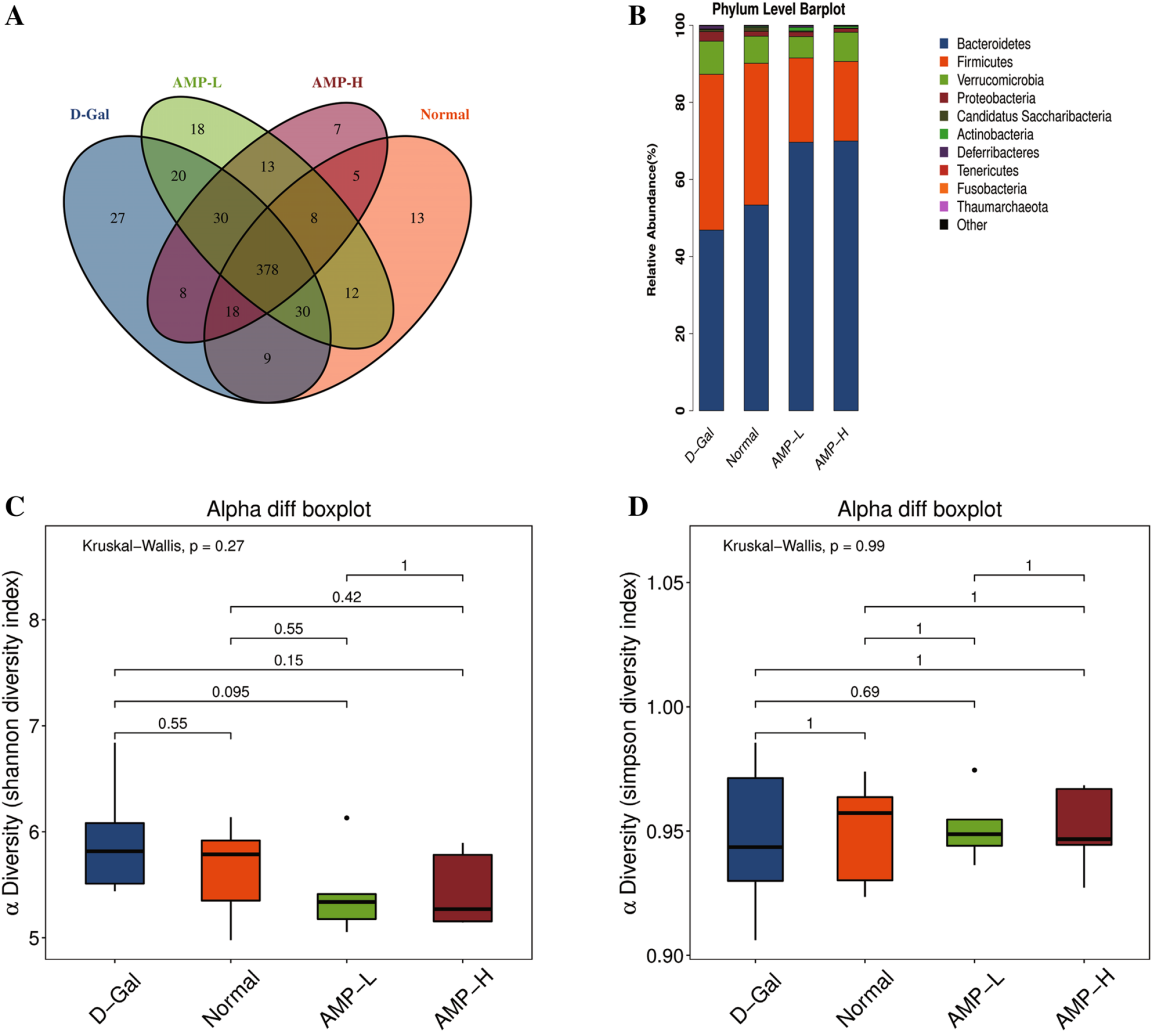

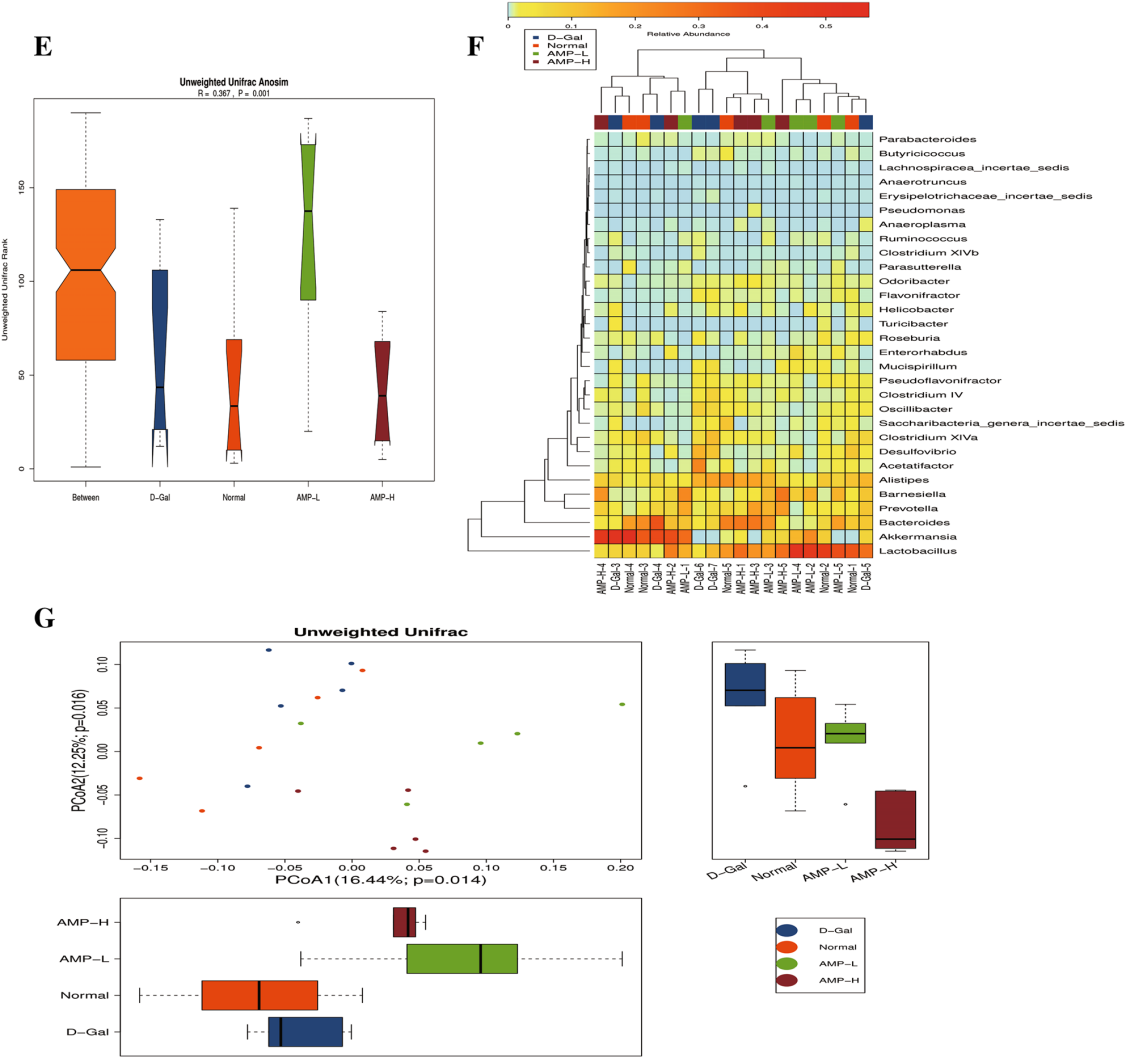
AMP improves D-Gal-induced aging by regulating the intestinal flora of mice. (**A**) Venn diagram; (**B**) Phylum lever barplot. (**C**,**D**) Alpha diff boxplot. (**E**) Unweighted UniFrac ANOSIM. (**F**,**G**) Relative abundance and unweighted Unifrac. Data are expressed as the mean ± standard deviation (S.D), n = 10. *p < 0.05 or **p < 0.01 vs. normal group; ^*#*^*p* < 0.05 or ^*##*^*p* < 0.01 vs. D-Gal group. OTUs were clustered using UPARSE version 7.1 (http://drive5.com/uparse/). The images were generated using R 3.6.3 (https://www.r-project.org/) and pheatmap package (https://cran.r-project.org/web/packages/pheatmap/).

The Venn diagram showed that the number of OTUs in the normal group, D-Gal group, AMP-L, and AMP-H groups were 473,520,509, and 467, respectively. There were 435 OTUs in the normal group and the D-Gal group. AMP-L, AMP-H group had a total of 458 and 434 OTUs, and the AMP-L and AMP-H group had a total of 429 OTUs (Fig. 6A). According to the species abundance table, the relative abundance of the *Bacteroides* and *Firmicutes* phyla was relatively increased in the four groups. After AMP treatment, the relative abundance of the *Bacteroides* phylum was increased, while the relative abundance of *Firmicutes* was decreased (Fig. 6B). The dominant bacterial groups significantly enriched in the four groups were *Lactobacillus*, *Akkermansia*, *Bacteroides*, *Prevotella*, *Barnesiella* and *Alistipes*, but there were no significant differences among the groups (Fig. 6F).

As shown in Fig. 6C,D, the α diversity (Shannon and Simpson index) increased from 0.27 to 0.99. The β diversity analysis based on weighted Unifrac clustering (pCoA, ANOSIM) showed that the difference between the four groups of mice was significantly greater than the difference within groups, and there were significant differences (R = 0.367, p = 0.001) (Fig. 6E). The pCoA diagram showed that although the flora in the four groups had changed by varying degrees, the flora of the AMP-L group was similar to the normal group of mice, while the flora of the AMP-H group was significantly different from the rest (Fig. 6G).

## Discussion

d-Galactose is a reducing sugar that can induce oxidative stress and tissue damage, lead to cell apoptosis, and mitochondrial dysfunction, and decrease antioxidant defense system function^37–39^. It is used on rodents to achieve a long-term preclinical aging model^43,44^. Substantial research has demonstrated that medicinal polysaccharides can effectively prevent senescence^45,46^. Meanwhile, *A. melancarpa* polysaccharides have only been reported for their anti-hypertensive and anti-atherosclerotic activity. We evaluated the anti-aging effect of AMP by measuring the body weight, organ index, and levels of MDA, SOD, and CAT, which were widely used as indicators of aging. Compared with D-Gal group, AMP treatment significantly increased the weight growth of aging mice, and their heart, liver, kidney, and brain all exhibited different levels of improvement. In addition, the eight-arm maze test was used to evaluate spatial work and reference memory in mice. The research showed that AMP significantly retarded aging in mice and the feeding distance and time of aging mice. The outward appearance observations, including fur, mental state and activity, and also demonstrated the protective effect of AMP on aging. H&E analysis showed that AMP treatment alleviated D-Gal-induced brain injury, and also improved neurological function.

The intestinal microbes are often called the “second genome.” They can affect the body during aging, including microbes, genomic DNA, proteins, and metabolites^47–49^. In aging mice, the diversity of intestinal biota decreased, with beneficial bacteria decreasing and facultative anaerobes increasing, which causes cognitive and memory functions to decline^43,44,50,51^. To determine whether AMP alleviated the aging state of the body by improving the mouse flora, we collected mouse feces and used these for 16S rDNA analysis. The proportion of beneficial bacteria in the Bacteroides was significantly increased. The β diversity analysis based on weighted Unifrac clustering (pCoA, ANOSIM) showed that the difference between the four groups (R = 0.367, p = 0.001) was significantly greater than the difference within groups. In summary, AMP had obvious effects on intestinal flora, which opens up a new area of aging research.

In the mechanism study, we considered the oxidative stress, inflammation, and apoptosis aspects, which were shown to be associated with aging. As a key factor in the regulation of bioenergy metabolism, AMPK plays a vital role in anti-inflammatory and growth regulation, which are also key to metabolic-related diseases^52,53^. AMPK is also related to multiple signaling pathways such as SIRT1, NF-κB, mTOR, and P53^54^. P53 tumor suppressor protein is a multifunctional transcription factor that regulates cell proliferation, cycle and apoptosis. When cells are damaged and become cancerous, p53 will induce cell aging and prevent them from further malignant transformation into tumor cells. This shows that the level of P53 will increase to age^55^, and our research also confirmed this phenomenon.

As a pivot protein, we focused on the role of the AMPK/SIRT1/NF-κB signaling pathway in relation to aging and the chronic inflammation associated with aging. The increased trans-regulatory activity of NF-κB complex with age and the association with chronic inflammation with increased NF-κB activity and metabolic diseases such as diabetes and atherosclerosis suggest the role of NF-κB signaling in the aging process^56^. Metformin, based on clinical trials in Europe, is an anti-aging agent, and an agonist for AMPK. It also inhibits the NF-κB signaling pathway for anti-inflammatory effects^57^. NF-κB promotes the transcription and translation of the NLRP3 inflammasome molecules, and then activates NLRP3 inflammasome in response to inflammasome activators, releasing inflammatory factors^58^. It combines with ASC, promotes the maturation of IL-1β inflammatory factors, and induces cell pyroptosis. Inflammatory bodies are accompanied by pyroptosis. For this reason, we also measured the expression levels of Caspase1 and GSDMD. GSDMD has also been called the “executioner" of pyroptosis^59^. The results illustrated that AMP had a significant inhibitory effect on inflammasome in the brain tissue of D-Gal-induced aging mice, thereby postponing the process of aging, which was consistent with the previous research results^60^. Interestingly, this study verified the result by examining the AMPK pathway that had an indirect regulatory effect of AMPK on NF-κB. The results may explain the decreased activity of AMPK with aging, resulting in an energy metabolism imbalance and inflammatory response^61^.

Oxidative stress is a key factor in the pathogenesis of neurodegenerative diseases^62^. Nrf2 is a key transcription factor regulating oxidative stress and exists in the cytoplasm under normal physiological conditions. Once activated, it enters the nucleus, regulates the expression of downstream proteins, and has an anti-inflammatory effect^63^. Heme oxygenase-1 (HO-1) is one of the most widely distributed antioxidant enzymes in the body, and it has anti-inflammatory effects^64,65^. We found that AMP treatment promoted the entry to Nrf2 Nuclear and up-regulated the expression of HO-1. We tested the indicators related to oxidation (MDA, SOD, CAT), and the results were the same as we expected. The PI3K/Akt/mTOR pathway is one of the most important upstream signals for regulating Nrf2 nuclear translocation and the expression of second-stage antioxidant enzymes^66–68^.We explored the PI3K/Akt/mTOR pathway-related proteins and tested the downstream apoptotic proteins. The apoptotic protein family, including Bax and Bcl-2, are typical regulators of cell proliferation and apoptosis^69^. In this study, AMP increased the expression of P-PI3K and P-AKT, inhibited the expression of Bax and Caspase 3, and enhanced the expression of Bcl-2 through the PI3K/AKT pathway. These data indicated that AMP significantly improved D-Gal-induced oxidative stress injury in aging brain tissue and inhibited cell apoptosis by regulating the PI3K/AKT/mTOR signaling pathway, and retarding the aging process.

## Conclusions

In conclusion, AMP was confirmed to be active in anti-aging by the basic characteristics and indicators of mice, behavioral space, learning, memory functions, and intestinal flora analysis. We analyzed its possible mechanism in oxidative stress, inflammation, apoptosis, and other aspects. AMP inhibited NLRP3 inflammasome by the AMPK/SIRT1/NF-κB signaling pathway and regulated the intestinal flora delay of aging in mice. These evidences revealed the potential mechanism of AMP for delaying aging. However, aging is a comprehensive manifestation of the whole body, and this study only detected the brain, and the impact on AMP on other organs remains further studies.

## Materials and methods

### Chemicals and materials

Aspartate aminotransferase (AST), alanine aminotransferase (ALT), reduced glutathione (GSH), superoxide dismutase (SOD), and malondialdehyde (MDA) threonine were obtained from Nanjing Jiancheng Bioengineering Institute Lignin-Eosin (H&E) commercial assay kits. D-Gal (purity ≥ 95%) was purchased from Sigma (St. Louis, Mo, USA). Western blot-related antibodies were purchased from Proteintech and Arigo (Changchun, China).All other chemicals and reagents used in the study were of analytical grade.

### Preparation of AMP

#### Separation and purification of aronia polysaccharides

We washed the fresh fruits and extracted the polysaccharides by decoction in distilled water. We concentrated the decoction to one-eighth of the original volume, and then added three times the volume of 95% ethanol to precipitate overnight in a refrigerator at 4 °C to obtain a precipitate. We added water to dissolve, and then alcohol precipitation was repeated three times to obtain the crude polysaccharides after defatting, depigmentation, and preliminary purification by macroporous resin D101, vacuum freeze-drying to constant weight, and recording as AMP. After preliminary purification using the macroporous resin, 1 g of AMP was dissolved in deionized water, and then deionized water (flow rate 1.0 mL/min) was passed through the DEAE-52 fiber column (3 × 40 cm) for elution. The diameter of DEAE-52 cellulose was 300 mesh. The eluate was collected by an automatic collector. Each test tube (10 mL) was collected every 10 min, for a total of 150 tubes. The polysaccharide in the eluate was detected by the sulfuric acid phenol method. We measured the absorbance value and drew an elution curve to determine the composition of the MWP. After vacuum freeze-drying, a fure light brown-yellow powder was obtained, which was used for subsequent structure identification.

#### Fourier-transformed infrared (FT-IR) spectroscopic analysis

The sample was weighed 2 mg, and the sample plus 200 mg of potassium bromide was pressed into tablets. The blank control tablets were pressed only using potassium bromide powder. The samples were scanned and recorded on a Fourier transform infrared spectrometer (JASCO FT/IR-620 spectrometer; JASCO, Hachioji, Japan).

#### Monosaccharide composition analysis

The monosaccharide composition of AMP was determined by ion chromatography. We heated 5 mg of sample and TFA at 121 °C for 2 h, dried with nitrogen, removed residual TFA with methanol, and repeated this three times.

The chromatographic system used the Thermo ICS5000^+^ ion chromatography system (ThermoFisher Scientific, USA), Dionex™ CarboPac™ PA10 (250 × 4.0 mm, 10 μm) liquid chromatography column; the injection volume was 20 μL, and column temperature was 30 °C; mobile phase A (H_2_O) and mobile phased B (100 mM NaOH).

Fucose (Fuc), rhamnose (Rha), arabinose (Ara), galactose (Gal), Glucose (Glc), xylose (Xyl), mannose (Man), Galacturonic acid (Gal-UA), Glucuronic acid (Glc-UA), and Mannuronic acid (Man-UA) were used as standard monosaccharides. The method of their treatment was the same as that used for sample analysis. The qualitative and quantitative analyses were scord according to the retention time of chromatographic peak.

### Analysis of the anti-aging activity of polysaccharide

#### Animals and experimental design

A total of 40 adult male ICR mice (6–8 weeks old, weight 22–25 g) were purchased from Changchun Yisi Experimental Animal Co., Ltd., with quality certificate SCXK (JI) 2019-0008 (Changchun, China). Mice were maintained under constant temperature and humidity in pathogen free conditions at a 12:12 h (L:D) photoperiod with unlimited access to food and water. All animal investigational processes were done according to the Guide for the Attention and usage of Laboratory Animals and permitted by the Animal Investigational Morals Committee of Jilin Agricultural University. The ethics approval number was 2019-08-28-001.

As shown in Fig. 2A, we have established the basic flow of experimental operations. After 3 weeks of adaptation, the mice were randomly divided into four groups: normal group, D-Gal group, AMP low-dose group and high-dose group, with 10 mice in each group. In the D-Gal group, the dose was increased by 200 mg/kg by intraperitoneal injection until the dose was increased to 1000 mg/kg to maintain the same dose, once per 3 days for 12 weeks, and AMP (100 and 200 mg/kg) was given daily by oral gavage after 6 weeks of D-Gal-induced. The same volume of saline was given to the normal group. At 24 h after the last dose, all mice were executed and dissected to obtain the blood supernatant, which was isolated after centrifugation at 4 °C for 10 min and stored at − 80 °C until analysis. We harvest brain, heart, kidney, spleen and liver tissues to calculate the organ index: organ weight index = organ weight (mg)/body weight (kg). We used physiological saline in a ratio at a 1:9 and ground tissue grinder at 4 °C. The separated homogenate supernatant was centrifuged at 4000×*g* at 4 °C for 10 min and then stored at − 80 °C to measure cholinergic function. Brain tissue samples were fixed in formalin for histological examination, and other sections were rapidly stored frozen in liquid nitrogen.

#### Tissue collection and sample preparation

The initial and final weights of mice were collected along with the feces of mice and immediately placed in liquid nitrogen and frozen for subsequent intestinal flora analysis. After fasting for 12 h, blood was collected by orbital blood collection and determination of basic oxidation indexes. The brain was immediately removed and stored at − 80 °C for biochemical analysis. Mouse organs were collected and weighed for organ index analysis.

### Biochemical analysis

Fresh blood was centrifuged at 3000 rpm, 4 °C for 30 min, serum was collected, and the contents of glutathione (GSH), superoxide dismutase (SOD), and malondialdehyde (MDA) were used to evaluate oxidation. The brains of mice were homogenized with 0.9% saline, and the acetylcholinesterase (AchE) content was determined to evaluate the damage of neurons in the brain.

### Histological analysis

After the mice were dissected, fresh brains of the mice were obtained, embedded in paraffin, and were detected using a hematoxylin and eosin (H&E) staining kit according to manufacturer instructions. The results were obtained using light microscope (Leica DM750, Germany).

### Western blot

A protein extraction kit (Thermo) was used to obtain brain supernatant. The samples were then separated on SDS polyacrylamide gel and transferred to a PVDF membrane, blocked with BSA for 1.5 h, washed with TBST for 5 min three times, and incubated with different antibodies overnight. The membrane was rinsed three times in TBST for 5 min each time and then incubated with the HRP-labeled antibody for 1 h. The band was detected by ECL chemiluminescence solution, and the protein expression was displayed by Bio-Rad VersaDoc 3000 Imaging System (Bio-Rad, Mississauga, ON, Canada). The original image was obtained in the Supplementary Information.

### Behavioral testing

Eight-arm maze was used to test the space and learning ability of mice. Before testing, we acclimated the mice for a fixed time to adapt to the environment. They were fasted for 12 h then tested for 10 min, and the four arms were randomly selected for sugar pill induction. We used RMT-100 analysis software (Chengdu Taimeng Software Co., Ltd.) to collect data and the motion trajectory parameters were recorded. We analyzed the distance moved by the mouse during the experiment, the incubation period, and calculated the time/total time for the mouse to pass each quadrant.

### 16S rDNA high-throughput sequencing

A total of twenty stool samples were randomly selected from all groups for intestinal flora analysis (five samples per group). High-throughput sequence of the 16S V4 region was used to analyze and compare the intestinal flora. Using Uparse software, based on the OTU threshold of 97% similarity, the sequences were clustered into operational taxonomies (OTU). Based on the sequence reads and OTU, the α diversity, β diversity and linear discriminant analysis effect size (LEfSe) were analyzed.

### Statistical analysis

Statistical analysis was carried out using SPSS statistical software. The numerical comparison was performed by ANOVA test for analyzing the differences between the two groups. The results are shown as the mean ± SD, and the significance level is defined as *p* < 0.05.

## Supplementary Information

Supplementary Information.

## Data Availability

We confirm the study is reported in accordance with ARRIVE guidelines (https://arriveguidelines.org).

## Abbreviations

AMP: *Aronia melanocarpa* polysaccharide
AMPK: Adenosine 5′-monophosphate (AMP)-activated protein kinase
SIRT1: Sirtuin 1
NF-κB: Nuclear factor kappa-B
Nrf2: Nuclear related factor-2
HO-1: Heme oxygenase-1
PI3K: Phosphatidylinositol 3-kinase
AKT: Protein kinase B
mTOR: Mammalian rapamycin target protein
H&E: Hematoxylin and eosin
Fuc: Fucose
Rha: Rhamnose
Ara: Arabinose
Gal: Galactose
Glc: Glucose
Xyl: Xylose
Man: Mannose
Gal-UA: Galacturonic acid
Glc-UA: Glucuronic acid
Man-UA: Mannuronic acid
AChE: Acetyl cholinesterase
GSH: l-glutathione
SOD: Superoxide dismutase
CAT: Catalase
GSDMD: Gasdermin D
IL-1β: Recombinant rat IL-1β
Bcl-2: B cell-lymphoma-2
Bax: B-associated X

## References

[1] Ding Q (2016). Antioxidant and anti-aging activities of the polysaccharide TLH-3 from Tricholoma lobayense. Int. J. Biol. Macromol..

[2] Lin L (2016). Antioxidative and renoprotective effects of residue polysaccharides from *Flammulina velutipes*. Carbohydr. Polym..

[3] Govindan S (2016). Antioxidant and anti-aging activities of polysaccharides from *Calocybe indica* var. APK2. Exp. Toxicol. Pathol..

[4] Baar MP (2017). Targeted apoptosis of senescent cells restores tissue homeostasis in response to chemotoxicity and aging. Cell.

[5] Shay JW (2016). Role of telomeres and telomerase in aging and cancer. Cancer Discov..

[6] Latorre-Pellicer A (2016). Corrigendum: Mitochondrial and nuclear DNA matching shapes metabolism and healthy ageing. Nature.

[7] Wang Y, Hekimi S (2015). Mitochondrial dysfunction and longevity in animals: Untangling the knot. Science.

[8] Sun N, Youle RJ, Finkel T (2016). The mitochondrial basis of aging. Mol. Cell.

[9] Kaushik S, Cuervo AM (2015). Proteostasis and aging. Nat. Med..

[10] Reznick RM (2007). Aging-associated reductions in AMP-activated protein kinase activity and mitochondrial biogenesis. Cell Metab..

[11] Fryer, L. G. D. & Carling, D. AMP-activated protein kinase and the metabolic syndrome. In *Biochemical Society Transactions*, Vol. 33, 362–366 (Biochem Soc Trans, 2005). 10.1042/BST0330362 15787607

[12] Qiang W, Weiqiang K, Qing Z, Pengju Z, Yi L (2007). Aging impairs insulin-stimulated glucose uptake in rat skeletal muscle via suppressing AMPKα. Exp. Mol. Med..

[13] Borodkina AV (2016). Tetraploidization or autophagy: The ultimate fate of senescent human endometrial stem cells under ATM or p53 inhibition. Cell Cycle.

[14] Giannakou ME, Partridge L (2004). The interaction between FOXO and SIRT1: Tipping the balance towards survival. Trends Cell Biol..

[15] Galluzzi L, Kepp O, Kroemer G (2010). TP53 and MTOR crosstalk to regulate cellular senescence. Aging.

[16] Castro JP, Wardelmann K, Grune T, Kleinridders A (2018). Mitochondrial chaperones in the brain: Safeguarding brain health and metabolism?. Front. Endocrinol..

[17] Wang L (2021). Oxidative stress in oocyte aging and female reproduction. J. Cell. Physiol..

[18] Zhong M (2020). TGase regulates salt stress tolerance through enhancing bound polyamines-mediated antioxidant enzymes activity in tomato. Environ. Exp. Bot..

[19] Harris IS, DeNicola GM (2020). The Complex Interplay between Antioxidants and ROS in Cancer. Trends Cell Biol..

[20] Ciesielski GL, Oliveira MT, Kaguni LS, Kaguni LS, Oliveira MTBT-TE (2016). Chapter eight—animal mitochondrial DNA replication. DNA Replication Across Taxa.

[21] De Gaetano A (2021). Mitophagy and oxidative stress: The role of aging. Antioxidants.

[22] Pan H (2021). Alginate oligosaccharide ameliorates d-galactose-induced kidney aging in mice through activation of the Nrf2 signaling pathway. Biomed. Res. Int..

[23] Chrubasik C, Li G, Chrubasik S (2010). The clinical effectiveness of chokeberry: A systematic review. Phytother. Res..

[24] Hukkanen AT, Pölönen SS, Kärenlampi SO, Kokko HI (2006). Antioxidant capacity and phenolic content of sweet rowanberries. J. Agric. Food Chem..

[25] Kokotkiewicz A, Jaremicz Z, Luczkiewicz M (2010). Aronia plants: A review of traditional use, biological activities, and perspectives for modern medicine. J. Med. Food.

[26] Tian Y (2017). Phenolic compounds extracted by acidic aqueous ethanol from berries and leaves of different berry plants. Food Chem..

[27] Vagiri M, Jensen M (2017). Influence of juice processing factors on quality of black chokeberry pomace as a future resource for colour extraction. Food Chem..

[28] Jin M, Zhao K, Huang Q, Xu C, Shang P (2012). Isolation, structure and bioactivities of the polysaccharides from *Angelica sinensis* (Oliv.) Diels: A review. Carbohydr. Polym..

[29] Zhao H (2016). The antihyperlipidemic activities of enzymatic and acidic intracellular polysaccharides by *Termitomyces albuminosus*. Carbohydr. Polym..

[30] Xie JH (2010). Isolation, chemical composition and antioxidant activities of a water-soluble polysaccharide from *Cyclocarya paliurus* (Batal.) Iljinskaja. Food Chem..

[31] YouGuo C, ZongJi S, XiaoPing C (2009). Evaluation of free radicals scavenging and immunity-modulatory activities of Purslane polysaccharides. Int. J. Biol. Macromol..

[32] Wang L, Liu HM, Qin GY (2017). Structure characterization and antioxidant activity of polysaccharides from Chinese quince seed meal. Food Chem..

[33] Martin GM (1983). Aging and cell structure. Hum. Pathol..

[34] Liu X (2020). Physicochemical characterization of a polysaccharide from *Agrocybe aegirita* and its anti-ageing activity. Carbohydr. Polym..

[35] Wang Y, Wei X, Jin Z (2009). Structure analysis of a neutral polysaccharide isolated from green tea. Food Res. Int..

[36] Pannese E (2011). Morphological changes in nerve cells during normal aging. Brain Struct. Funct..

[37] Kumar A, Prakash A, Dogra S (2010). Naringin alleviates cognitive impairment, mitochondrial dysfunction and oxidative stress induced by d-Galactose in mice. Food Chem. Toxicol..

[38] Anand KV, Mohamed Jaabir MS, Thomas PA, Geraldine P (2012). Protective role of chrysin against oxidative stress in d-Galactose-induced aging in an experimental rat model. Geriatr. Gerontol. Int..

[39] Lu J (2006). Quercetin reverses d-Galactose induced neurotoxicity in mouse brain. Behav. Brain Res..

[40] Penley SC, Gaudet CM, Threlkeld SW (2013). Use of an eight-arm radial water maze to assess working and reference memory following neonatal brain injury. J. Vis. Exp..

[41] Clelland CD (2009). A functional role for adult hippocampal neurogenesis in spatial pattern separation. Science.

[42] Soellner DE, Grandys T, Nuñez JL (2009). Chronic prenatal caffeine exposure impairs novel object recognition and radial arm maze behaviors in adult rats. Behav. Brain Res..

[43] Choi J, Hur T-Y, Hong Y (2018). Influence of altered gut microbiota composition on aging and aging-related diseases. J. Lifestyle Med..

[44] Kelly JR, Minuto C, Cryan JF, Clarke G, Dinan TG (2017). Cross talk: The microbiota and neurodevelopmental disorders. Front. Neurosci..

[45] Ahlemeyer B, Krieglstein J (2003). Neuroprotective effects of Ginkgo biloba extract. Cell. Mol. Life Sci..

[46] Bastianetto S, Quirion R (2002). Natural extracts as possible protective agents of brain aging. Neurobiol. Aging.

[47] Mangiola F, Nicoletti A, Gasbarrini A, Ponziani FR (2018). Gut microbiota and aging. Eur. Rev. Med. Pharmacol. Sci..

[48] Qin J (2010). A human gut microbial gene catalogue established by metagenomic sequencing. Nature.

[49] Carding S, Verbeke K, Vipond DT, Corfe BM, Owen LJ (2015). Dysbiosis of the gut microbiota in disease. Microb. Ecol. Health Dis..

[50] Saulnier DM (2013). The intestinal microbiome, probiotics and prebiotics in neurogastroenterology. Gut Microbes.

[51] Gareau MG (2016). Cognitive function and the microbiome. International Review of Neurobiology.

[52] Ke R, Xu Q, Li C, Luo L, Huang D (2018). Mechanisms of AMPK in the maintenance of ATP balance during energy metabolism. Cell Biol. Int..

[53] Sekar P, Huang DY, Hsieh SL, Chang SF, Lin WW (2018). AMPK-dependent and independent actions of P2X7 in regulation of mitochondrial and lysosomal functions in microglia. Cell Commun. Signal..

[54] Caldeira CA (2021). Resveratrol: Change of SIRT 1 and AMPK signaling pattern during the aging process. Exp. Gerontol..

[55] Lee DH, Lee TH, Jung CH, Kim YH (2012). Wogonin induces apoptosis by activating the AMPK and p53 signaling pathways in human glioblastoma cells. Cell. Signal..

[56] Yeung F (2004). Modulation of NF-κB-dependent transcription and cell survival by the SIRT1 deacetylase. EMBO J..

[57] Salminen A, Hyttinen JMT, Kaarniranta K (2011). AMP-activated protein kinase inhibits NF-κB signaling and inflammation: Impact on healthspan and lifespan. J. Mol. Med..

[58] Boaru SG, Borkham-Kamphorst E, Tihaa L, Haas U, Weiskirchen R (2012). Expression analysis of inflammasomes in experimental models of inflammatory and fibrotic liver disease. J. Inflamm..

[59] Shi J (2015). Cleavage of GSDMD by inflammatory caspases determines pyroptotic cell death. Nature.

[60] Fehervari Z (2017). Inflammasomes in human aging. Nat. Immunol..

[61] Hu R (2020). Salidroside ameliorates endothelial inflammation and oxidative stress by regulating the AMPK/NF-κB/NLRP3 signaling pathway in AGEs-induced HUVECs. Eur. J. Pharmacol..

[62] Chiurchiù V, Orlacchio A, Maccarrone M (2016). Is modulation of oxidative stress an answer? The state of the art of redox therapeutic actions in neurodegenerative diseases. Oxid. Med. Cell. Longev..

[63] Ou Z (2018). Metformin treatment prevents amyloid plaque deposition and memory impairment in APP/PS1 mice. Brain. Behav. Immun..

[64] Li J (2018). Mkp-1 cross-talks with Nrf2/Ho-1 pathway protecting against intestinal inflammation. Free Radic. Biol. Med..

[65] Huang B (2018). α-Cyperone inhibits LPS-induced inflammation in BV-2 cells through activation of Akt/Nrf2/HO-1 and suppression of the NF-κB pathway. Food Funct..

[66] Chen HH, Chen YT, Huang YW, Tsai HJ, Kuo CC (2012). 4-Ketopinoresinol, a novel naturally occurring ARE activator, induces the Nrf2/HO-1 axis and protects against oxidative stress-induced cell injury via activation of PI3K/AKT signaling. Free Radic. Biol. Med..

[67] Gao W (2018). Glutathione homeostasis is significantly altered by quercetin via the Keap1/Nrf2 and MAPK signaling pathways in rats. J. Clin. Biochem. Nutr..

[68] Xu J (2017). Garcinia xanthochymus extract protects PC12 cells from H_2_O_2_-induced apoptosis through modulation of PI3K/AKT and NRF2/HO-1 pathways. Chin. J. Nat. Med..

[69] Sadhukhan P, Saha S, Dutta S, Sil PC (2018). Mangiferin ameliorates cisplatin induced acute kidney injury by upregulating Nrf-2 via the activation of PI3K and exhibits synergistic anticancer activity with cisplatin. Front. Pharmacol..

