# Supplementary material for: Aronia melanocarpa polysaccharide ameliorates inflammation and aging in mice by modulating the AMPK/SIRT1/NF-κB signaling pathway and gut microbiota
Source: Sci Rep. 2021 Oct 18;11:20558. doi: 10.1038/s41598-021-00071-6 (PMC8523697; doi:10.1038/s41598-021-00071-6)

Bax


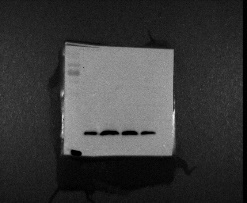


BCL-2


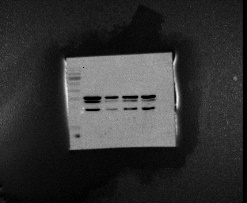


Caspase3


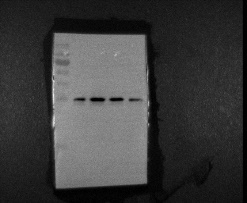


Actin


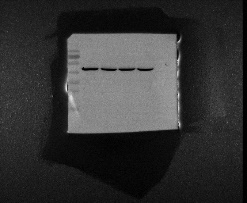


GSDMD


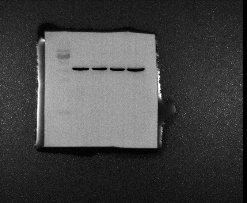


IL-1β


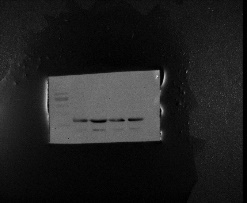


NF-κB


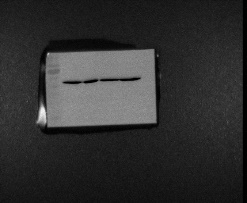


p-NF-κB


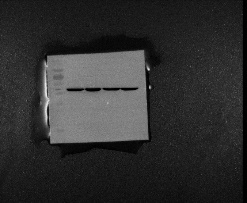


IκBα


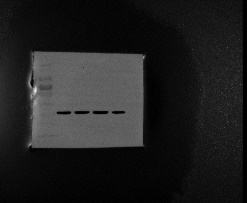


p-IκBα


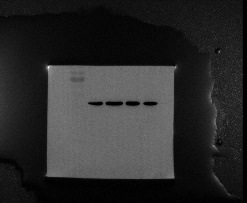


Actin


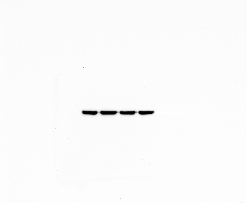


PI3K


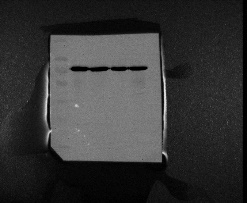


p-PI3K


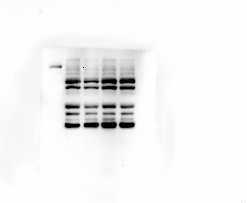


AKT


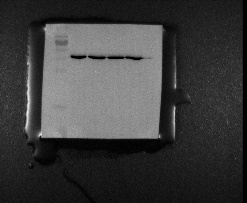


p-AKT


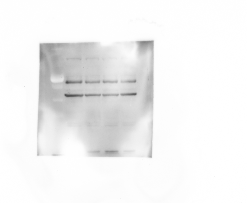


mTOR


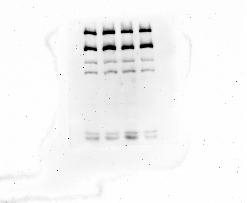


p-mTOR


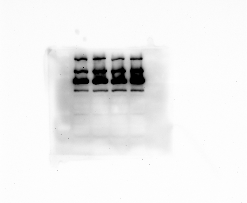


Actin


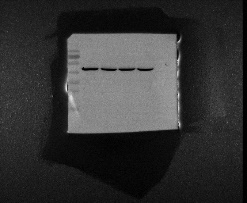


P53


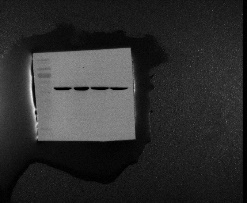


AMPK


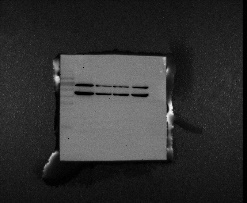


SIRT1


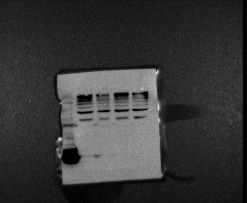


ASC


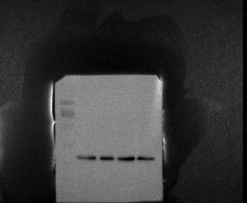


Caspase1


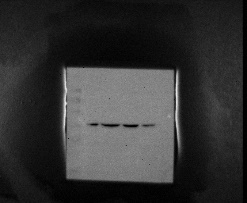


NALP3


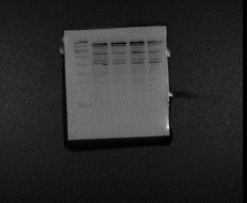


Actin


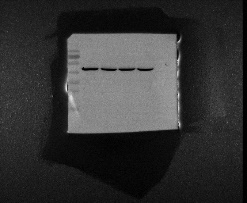


HO-1


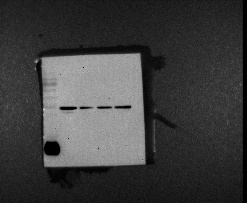


Laminβ1


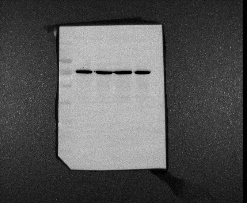


Nrf2


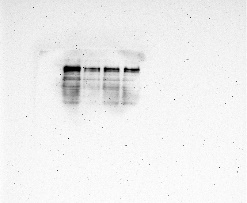

Supplement: Supplementary file 1 — Supplementary Information. [file 41598_2021_71_MOESM1_ESM.docx]
